# Spectral Characterization of Hierarchical Network Modularity and Limits of Modularity Detection

**DOI:** 10.1371/journal.pone.0054383

**Published:** 2013-01-28

**Authors:** Somwrita Sarkar, James A. Henderson, Peter A. Robinson

**Affiliations:** 1 School of Physics, University of Sydney, Sydney, New South Wales, Australia; 2 Brain Dynamics Center, Sydney Medical School – Western, University of Sydney, Westmead, New South Wales, Australia; Universidad de Zarazoga, Spain

## Abstract

Many real world networks are reported to have hierarchically modular organization. However, there exists no algorithm-independent metric to characterize hierarchical modularity in a complex system. The main results of the paper are a set of methods to address this problem. First, classical results from random matrix theory are used to derive the spectrum of a typical stochastic block model hierarchical modular network form. Second, it is shown that hierarchical modularity can be fingerprinted using the spectrum of its largest eigenvalues and gaps between clusters of closely spaced eigenvalues that are well separated from the bulk distribution of eigenvalues around the origin. Third, some well-known results on fingerprinting non-hierarchical modularity in networks automatically follow as special cases, threreby unifying these previously fragmented results. Finally, using these spectral results, it is found that the limits of detection of modularity can be empirically established by studying the mean values of the largest eigenvalues and the limits of the bulk distribution of eigenvalues for an ensemble of networks. It is shown that even when modularity and hierarchical modularity are present in a weak form in the network, they are impossible to detect, because some of the leading eigenvalues fall within the bulk distribution. This provides a threshold for the detection of modularity. Eigenvalue distributions of some technological, social, and biological networks are studied, and the implications of detecting hierarchical modularity in real world networks are discussed.

## Introduction

Many real world networks have been reported to have modular or hierarchical modular organization, including social networks [1], collaboration networks [1], biological networks such as structural and functional brain networks [2]–[5], metabolic networks [6], and gene expression networks [7], and technological networks such as the Internet, the World Wide Web, and the global air transportation network [1]. Reliably detecting the hierarchical and modular organization of complex systems provides us with a way to understand how their microscale structure scales up to the macroscale, and how the system is able to perform specific behaviors and functions.

Despite the importance of hierarchy and modularity, there exists no algorithm-independent way to characterize how “hierarchically modular” a network is. Since detection of modularity is dependent upon the assumptions made in specific modularity detection algorithms, these assumptions significantly affect the results. For example, a modularity detection algorithm that is based on strict graph partitioning techniques will fail to find overlaps between communities and hierarchical organization, unless specifically modified. In addition, there are many algorithms that will find optimal partitions in networks with no modularity. For example, an algorithm that is designed to locate optimal partitions will do so even for nonmodular networks. Second, many of the algorithms are based on optimizing the modularity metric *Q* [1], [8], which is computed for a particular division of a network into communities by comparing this division to that of a null reference model – a random graph with the same size and degree distribution, but no community structure. The modularity metric *Q* has been shown to suffer from a resolution limit problem, meaning that it cannot detect the smallest size communities relative to network size [9]. Further, the computation of *Q* requires that the network first be divided into modules before it can be evaluated, and provides no information on the uniqueness of the postulated modules; i.e., which solution should be preferred if two solutions have the same *Q* value. Further, no such well accepted metric exists for measurement of hierarchical modularity in networks, although there exist some modularity detection algorithms based on quantifying the quality of hierarchical modularity and partitions in network structure [10].

In this paper, the main results are a set of methods to address the above gap. We present an algorithm-independent manner of characterizing network modularity. We use results from random matrix theory and spectral graph theory to derive the spectrum of eigenvalues for hierarchically modular networks generated using a stochastic block model and show that the spectrum contains clear fingerprints of hierarchical modularity. Further, we rederive some known results about the spectra of modular networks, which are simply shown to be a special case of hierarchically modular networks with a single hierarchical level. Using the spectral results, we empirically derive the limits of modularity detection; i.e., a way to compare the degree of modularity that actually exists in the network, versus its actual detectability, by varying the degree of probabilities of instantiating edges at various hierarchical levels. It is shown that even when modularity and hierarchical modularity are present in a sufficiently weak form in the network, it is impossible to detect them, because some of the leading eigenvalues fall within the bulk distribution of eigenvalues around the origin and are no more separated from it. The point at which this happens is estimated in terms of the edge instantiation probabilities, and sets a threshold beyond which modularity cannot be detected even when present in the network. Eigenvalue distributions of some technological, social, and biological networks are studied, and the implications of detecting hierarchical modularity in real world networks are discussed.

Some previous work [9] and a very recent study [11] has shown a similar result for modular networks (a subcase in our work), but not hierarchically modular networks, thereby making the results in this paper more general. They derived analytical results for the threshold of modularity detection in undirected, modular graphs. Our findings agree with their results, but our results in this paper are valid for both directed and undirected graphs, and we include hierarchical modularity. Modular networks are shown as a special case of the general framework. The work in [11] asserted that the spectral signatures of modularity detection are optimal in the sense that no other method can detect modularity in a regime where the spectral methods fail. This establishes that the results we present in this paper on the limits of modularity detection are general in the sense that if the spectral fingerprint fails to detect weak forms of modularity in a network, then any of the current methods and algorithms using spectral approaches for modularity detection are likely to be unable to detect it.

## Results

In this section, we derive and illustrate the methods that constitute the main results of this paper. Our main results are (i) derivation of the spectrum of hierarchically modular graphs; i.e., the mean expected values of the largest eigenvalues of the adjacency matrix of the graph, (ii) establishing the limits of “how modular” a real world system is through a study of the properties of the spectrum and its distribution, and (iii) establishing the limits of detection of hierarchical modularity and modularity as permitted by the spectral approach; i.e., given the amount or degree of modularity, how much of this modularity can (or cannot) be detected using the spectral approach.

Thus, in this work, we characterize the hierarchical modularity of a network in an algorithm-independent manner. The spectrum of modular networks with no hierarchy is shown to be a special case of the framework, and some known results on the spectrum of modular networks are thus automatically reproduced, thereby providing a unified basis to characterize network modularity in general. Finally, we empirically show that when probability parameters for instantiating edges in networks are varied, there is a threshold set by the probabilities and the limits of the bulk distribution of eigenvalues around the origin beyond which hierarchical modularity and modularity cannot be detected even if present.

### Spectrum of Hierarchically Modular Networks

We follow a typical stochastic block model form for contructing a hierarchical network, similar to [12]. This process involves construction of a hierarchically modular network by recursively placing random matrix blocks with decreasing levels of connecitivity between nodes in hierarchical levels in a block diagonal form. We consider the matrix
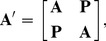
(1)where **A** is a random network of size *s* and edge probability *p*, and **P** is a random network of size *s* and edge probability *pq*. Here, the parameter *q* sets the level of decrease in connectivity between the various hierarchical levels. That is, *q* is a numeric parameter which is varied to define the connectivity of the first level hierarchy off-diagonal blocks or networks represented by **P**. For example, if 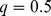, then the connectivity in **P** is 50% of the connectivity in **A**. If 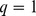, the network will no longer be hierarchical, but will simply be a random network of size 2*s* with connection probability *p* (since, in this case, 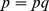). It is clear from the formulation that lower the value of *q*, stronger the hierarchical modular structure, and higher the value of *q* (to 1), weaker the hierarchical modular structure. This point is important in the following section on establishing the limits of detection of modularity. We know from the random matrix theorems established in *Methods* that the expected value of the largest eigenvalue of **A** is *sp* and that of **P** is *spq*. We thus rewrite **A′** as a sum of deterministic matrices **A** *^E^* and **P** *^E^* with entries 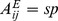 and 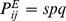, respectively, and a matrix of fluctuations around these means, obtaining



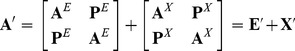
(2)The spectrum of 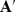 can now be decribed by independently describing the spectra of matrices 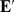 and 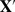.

First, it can be easily proved that the deterministic matrix 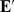 has the eigenvalue distribution
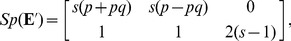
(3)and it gives us the mean expected values of the largest two eigenvalues and the mean value of rest of the bulk distribution of eigenvalues of 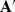. Consider a vector of the form 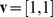, where the 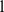 vectors have 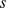 entries each. Then,



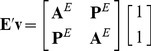
(4)

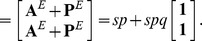


Thus, 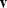 is an eigenvector of 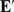 with the eigenvalue 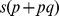. Similarly, consider vectors of the form 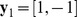 and 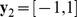, again with vectors 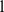 and 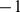 of size 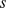. Then, by similar reasoning as above, 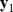 and 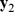 can be shown to be eigenvectors of 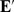 with eigenvalue 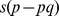. The other eigenvalues will all be 0.

Next we consider the spectrum of the fluctuation matrix 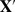. The expectation value of the entries of 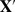 is 0 by definition, since 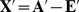. Thus, by Eq.(20), (see *Methods*) the spectrum of 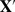 has a zero mean value, and all its eigenvalues are bounded by the spread limit 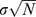, where 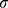 stands for the standard deviation of the values in 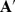. This 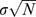 gives us the spread of the bulk distribution of 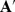 with zero mean.

**Figure 1:**
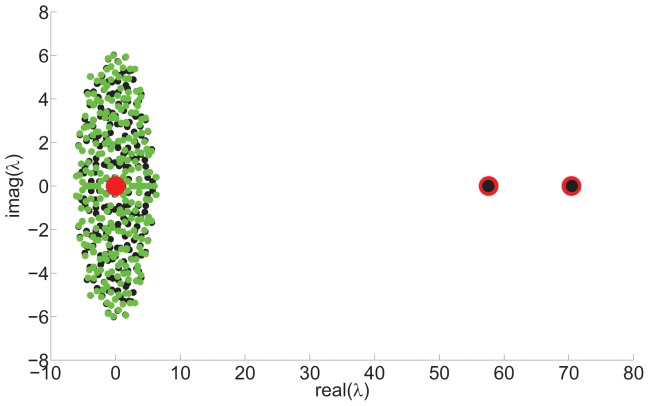
First level hierarchical network spectrum. 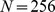
 node network, 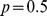, 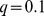, Black circles (below green circles) show actual spectrum. Red circles, centred on the point, show analytic prediction of mean expected eigenvalues from Eq. (3). Green circles show distribution of eigenvalues of the fluctuation matrix.

Thus, putting together the above results, the spectrum of 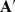 is
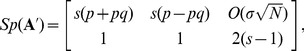
(6)showing that there will be two large eigenvalues separated from a bulk distribution of eigenvalues around the origin. Figure 1 shows examples of the actual eigenvalues and analytical predictions by Eq. (3) along with the actual eigenvalues of the fluctuation matrix.

We now define the second level of perturbation, where
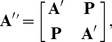
(7)where 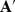 is the matrix defined in Eq. (1) and 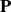 is a random network or matrix of size 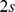 and edge probability 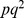. Note here the second hierarchical level: 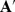 already has the first level of hierarchy built in as described previously, with the first level off-diagonal blocks having connectivity 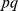 and the diagonal blocks having connectivity 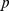, with 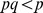. Now, the second level off-digonal blocks, represented by matrix 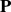, have connectivity 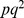 with 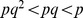. In general, the matrix 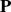 defines each successive level 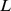 of perturbations of increasing size (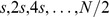) and decreasing probability of connection (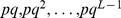), producing an extra level of hierarchical modular structure with each perturbation level.

Once again, we define this matrix 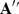 as a sum of a deterministic matrix and a fluctuation matrix in a form similar to described above, 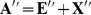. The mean expected values of the eigenvalues of 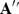, using similar analysis as before, are shown to be
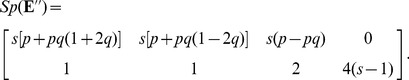
(8)


In general, for 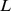 hierarchical levels, the expectation values of the eigenvalues of a hierarchical network 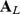, along with their multiplicities, are
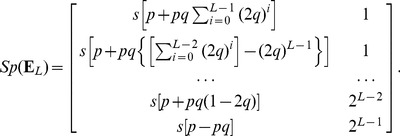
(9)


**Figure 2:**
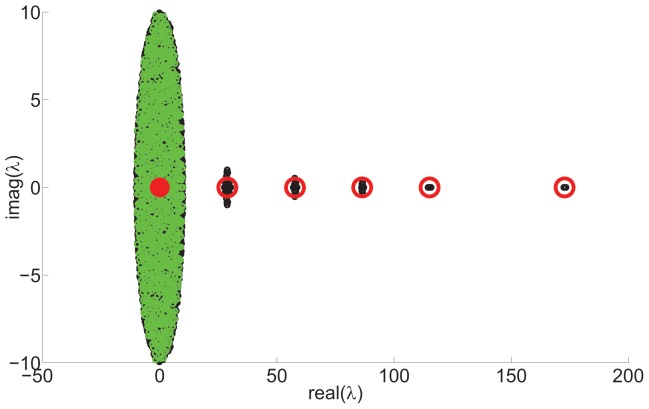
Full hierarchical network spectrum. 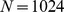
 node network, 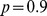, 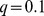. Black circles (below green circles) show actual spectrum. Red circles, centred on the point, show analytic prediction of mean expected eigenvalues from Eq. (9). Green circles show distribution of eigenvalues of the fluctuation matrix.

Figure 2 shows the spectra of 100 hierarchical networks, the eigenvalues of the fluctuation matrices, and the analytically predicted mean expected values for the largest eigenvalues for a network with 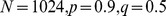 and 5 hierarchical levels with 1024 nodes at the coarsest level, followed by 2 clusters of 512 nodes, 4 clusters of 256 nodes, 8 clusters of 128 nodes, and 64 clusters of 16 nodes. Note that the spectrum and the predicted mean expected values of the 16 largest eigenvalues echo this pattern: there are 5 clusters of eigenvalues separated with large gaps, with the largest 2, 4, 8, and 16 eigenvalues in each cluster.

### Spectrum of Perturbed Modular, Nonhierarchical Networks

It is known that if a modular network has 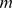 modules, then its spectrum will show 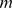 large eigenvalues [13], [14]. This result is easily rederived as a special case of the above framework. We consider an unperturbed modular network 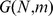 with 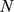 nodes and 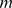 equally sized disconnected modules that are random networks of size 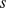 and nodes connected with probability 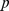, as defined in the previous section. The adjacency matrix 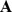 for this network has 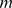 random blocks on the diagonal.

We now perturb this ideal modular network with block matrices of size 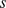, each of which is random network of 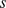 nodes with probability 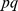 of an edge between two nodes, where 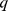 sets a rate of decrease in probability of an edge between two nodes. As above, we call these the perturbation matrices 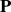. The perturbed modular network is represented by matrix 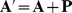, where 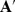 has the block form
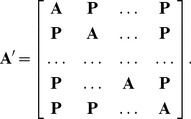
(10)


Thus, instead of considering higher powers of 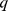 to set the levels of decrease of connectivity, we set q to a single value to produce a single levelled perturbed modular network. Using Eq. (9), and substituting the correct values for 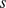, 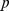, and 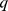, it can be easily shown that the spectrum of the perturbed modular network 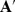 is
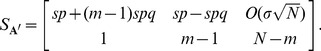
(11)


**Figure 3:**
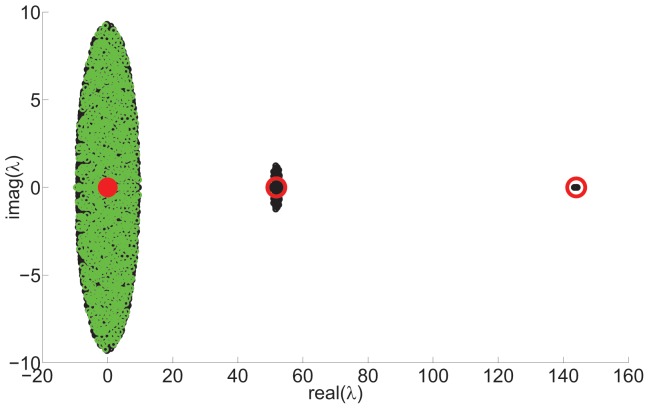
Full modular non-hierarchical network spectrum. 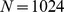
 node network, 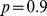, 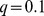. Black circles (below green circles) show actual spectrum. Red circles, centred on the point, show analytic prediction of mean expected eigenvalues from Eq. (11). Green circles show distribution of eigenvalues of the fluctuation matrix.

The largest eigenvalue of the perturbed matrix has a mean expected value of 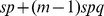 and the next 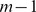 largest eigenvalues have a mean expected value of 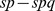. Figure 3 shows the actual, predicted, and fluctuation matrix eigenvalue distributions for a non-hierarchical modular network.

### Empirical Limits of Modularity Detection

From the above sections we see that the mean expected eigenvalues depend upon the network size 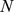, the number of modules 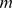, the size of each module 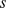, and the probability parameters 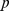 and 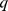. We vary all these parameters, and especially 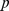 and 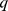 for given 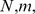 and 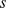, to explore how the mean expected eigenvalues vary. It is clear that the gaps between the largest eigenvalues and the bulk of the distribution provides us with the capacity to detect community structure. Therefore, at the point where the principal or largest eigenvalues are no longer separated from the bulk distribution is also the point where we lose the capacity to detect the community structure. It might be expected that this point will occur when 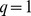, thereby making the probability of edges outside modules equal to that for those inside modules; i.e., 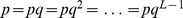. However, using the spectral results above, we find that this is not the case. We find that even when modularity is present in a weak form in the network, it will not be possible to detect it. Thus, for a given 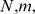 and 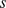, certain values of 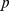 and 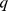 provide a detectability threshold beyond which it is not possible to detect modularity structure in networks, even if some modularity is present. We present the empirical location of this threshold for any network, in terms of the values of 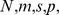 and 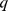. Very recently, a study has found similar results for modular networks [11], but we know of no other such studies for hierarchically modular networks. The study in [11] also asserts and demonstrates that if spectral modularity detection methods fail to detect community structure then no other method will detect it.

#### Limits of modularity detection in modular networks

**Figure 4:**
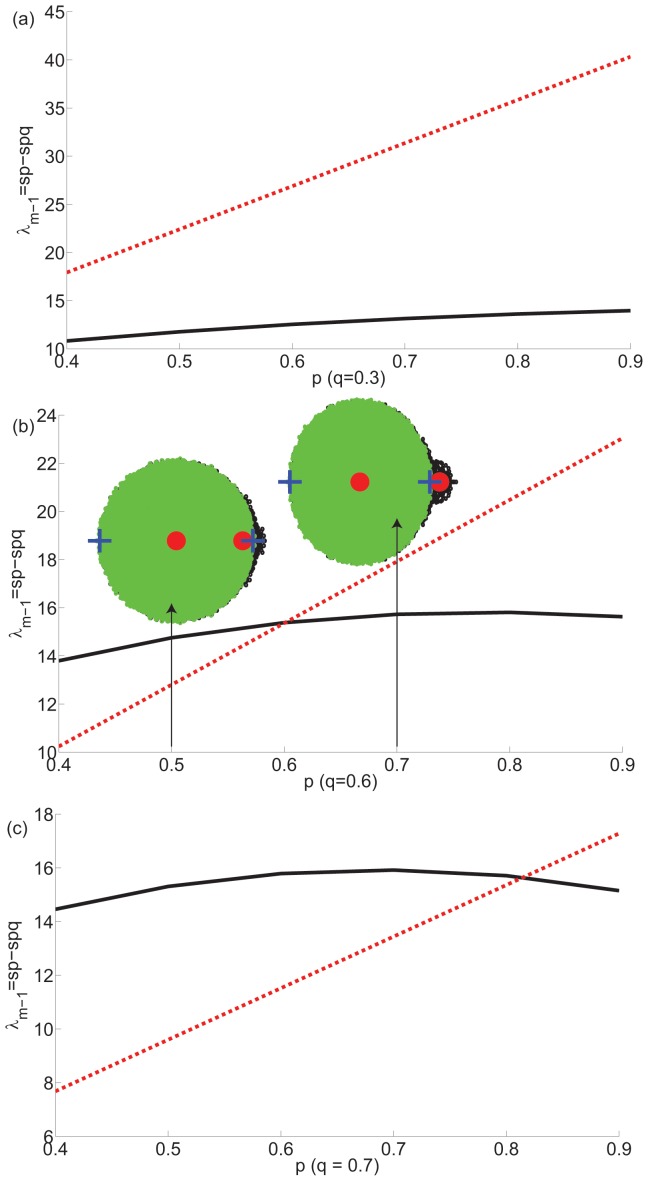
Limits of modularity detection in modular networks. Expectation value of second largest eigenvalues 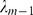 (red lines) and limits of bulk distribution 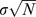 (black lines) are plotted for values of 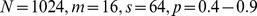 and (a) 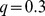, (black line below red line: modularity can be detected for all 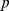), (b) 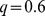, (modularity can only be detected for 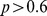, i.e., region with black line below red line), (c) 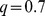 (modularity can only be detected for 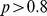, i.e., region with black line below red line). Threshold of detection is the point where the two lines cross. In (b), eigenvalue distributions are plotted for 100 networks at 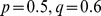 and 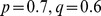. Black circles: actual eigenvalues, green circles: eigenvalues of fluctuation matrix, red circles: analytically predicted expectation values of second largest and bulk distribution of eigenvalues, blue pluses: predicted limits of bulk distribution of fluctuation matrix. Before threshold point, second largest eigenvalue falls within the bulk distribution, after threshold point, it falls outside the bulk distribution.

In a modular network, the mean expected value of the largest eigenvalue 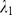 is 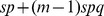, that of the second largest eigenvalue 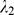 is 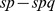, and the limits of the bulk distribution (all the other eigenvalues are denoted as 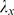), are 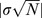, where 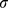 is the standard deviation of the entries in the fluctuation matrix as described in the previous section. The difference between 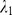 and 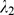 is
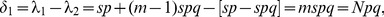
(12)which grows when 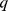 is increased relative to 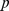 because 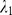 will grow and 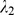 will become smaller as 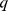 increases. The difference between 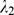 and the limits of the bulk distribution is 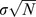




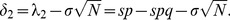
(13)As 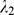 gets smaller with increasing 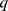, and the limits of the bulk distribution grow larger with increasing 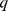, the point at which 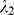 falls within the limits of the bulk distribution it will no longer be possible to detect the modularity structure. At 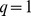, 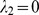, because 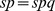. Since 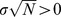, this point falls when 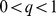, which implies that even when weak modularity is present in the network, it is no longer possible to detect it. This is seen in Figure 4 that shows 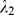 for chosen values of 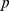 and 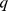 for given 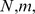 and 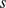. Thus, the condition that
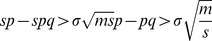
(14)provides the criterion for modularity detection, with the threshold given by the condition 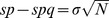. When the condition is violated, it will be impossible to detect modularity structure even when present. For example, in Fig. 4(b), it is easily observed that at 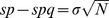, some weak modularity is present, but cannot be detected.

Equation (14) also shows that as the number of modules 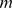 increases, and the size of modules 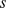 decreases, for a certain 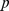, this threshold is violated for smaller and smaller values of 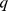. On the other hand, when the size of modules 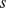 is larger, the number of modules 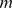 grows smaller, larger values of 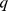 will not violate the threshold condition. Qualitatively, this implies that as network size increases, it gets harder to detect the smallest modules in the network.

#### Limits of modularity detection in hierarchical networks

**Figure 5:**
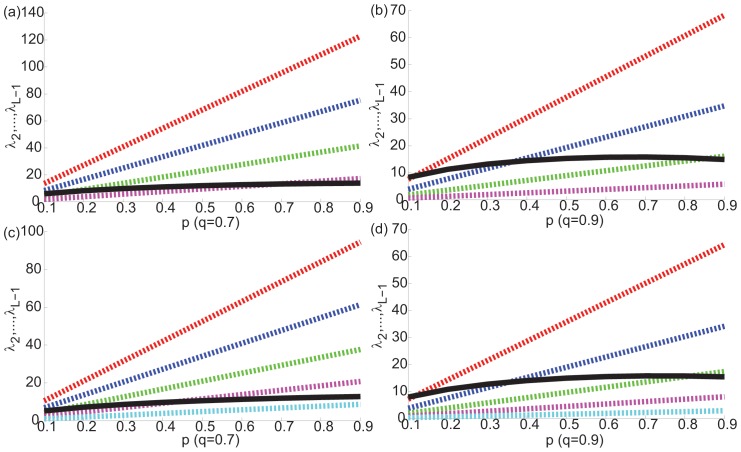
Limits of modularity detection in hierarchically modular networks. Mean expected value of 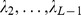 (red, blue, green, purple, cyan lines, respectively) and limits of bulk distribution 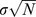 (black lines) are plotted for a 4 level hierarchical network with 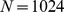, 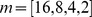, 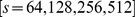, 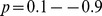 and (a) 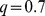 and (b) 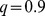, and for a 5-level hierarchical network 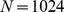, 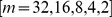, 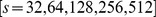, 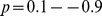 and (c) 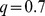 and (d) 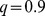. The points where the black lines cross the colored lines show thresholds of detection for the corresponding hierarchical levels. Modularity can only be detected above the black lines. Comparing (a) and (b), as 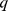 increases, the number of colored lines falling below the black lines increases. Comparing (b) and (d), as the number of hierarchical levels increase and module sizes at finer hierarchical levels become smaller, at the same 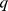 value, a larger number of colored lines fall below the black lines.

We recall that in a hierarchically modular network, the mean expected values of the largest eigenvalues are given by Eq. (9), and the limits of the bulk distribution (all the other eigenvalues are denoted as 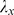), are described by 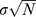, where 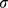 is the standard deviation of the entries in the fluctuation matrix as described in the previous section. From the spectrum we can see that the largest eigenvalue 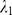 grows with increasing 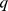, while the subsequent eigenvalues outside the bulk distribution get smaller. Further, the limits of the bulk distribution of the fluctuation matrix will also grow with increasing 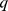. At the point where the leading eigenvalues fall within the limits specified by the bulk distribution of eigenvalues, it will be no longer possible to detect the corresponding hierarchical levels or the modules at these levels. These threshold conditions, starting from the cluster of eigenvalues signifying the finest hierarchical level in the network, are given by
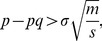
(15)

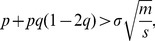
(16)and in general



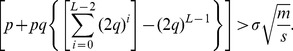
(17)
Figure 5 shows 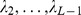 for chosen values of 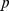 and 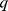 for given 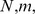 and 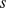. The points at which the threshold conditions stated above are violated, it is impossible to detect the modularity structure present in the network. Specifically, in the hierarchical case, the number of eigenvalue clusters that fall within the bulk distribution equals the number of hierarchical levels and modules at these levels that go undetected. For example, in Fig. 5(c), it is easily observed that for 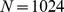 and 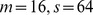 at the finest hierarchical levels, with 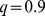, the first two (finest) hierarchical levels cannot be detected at any value of 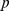, even though hierarchical modularity is present.

From the threshold conditions, it is also seen that as the sizes of modules 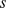 get smaller and the number of modules 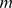 higher relative to network size 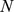, it will get harder and harder to detect the smallest sized modules at the finest levels of hierarchy for smaller and smaller values of 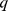. For example, in Fig. 5(f), the first 3 hierarchical levels cannot be detected, because the smallest module size is 32 instead of 64, as in Fig. 5(c). Thus, while the overall large scale hierarchy is detectable, finer levels of hierarchy signified by the eigenvalues that fall within the bulk distribution of eigenvalues lie undetected. As we show in the next section, this observation has significant implications for detection of modularity in real world networks that are known to have extraordinary complexity and multiscale levels of organization, such as human brain networks.

### Real World Networks

#### Evolving peer to peer internet networks

Peer-to-peer or P2P networks are decentralized, self-organized systems, in which individual computers connect to each other and communicate directly for the purposes of sharing information and resources, without dedicated or centralized servers [15]. Though these systems are guided by common goals (for example, of sharing CPU cycles and storage space), there is no central guiding authority. The resulting network topology and the dynamics of communication occurring on it are emergent; i.e., individual users interacting locally with other users determine the local decisions, but the large scale system behavior cannot be determined trivially from the local interactions alone. The highly decentralized self-organized nature of these evolving networks ensures large fluctuations in network size and numbers of edges, as the size and resulting topology of the network are completely determined by how many individual users are joining and leaving the network. Since many self-organized systems in nature and society show modular organization, we were interested in looking at the modularity properties of large scale evolving peer to peer networks, and to chart how modular organization of a guided self-organized system evolves dynamically over time.

**Figure 6:**
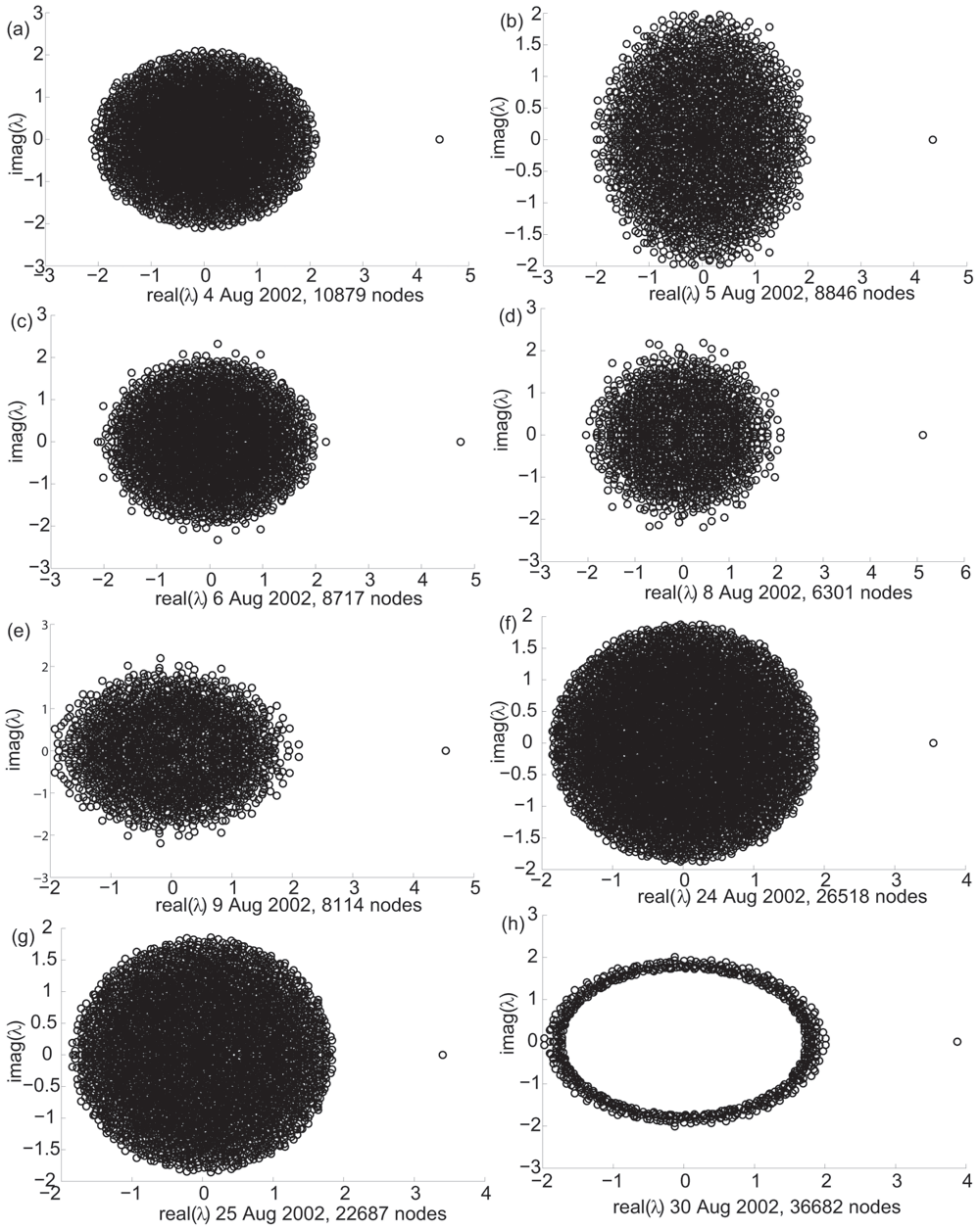
Gnutella evolving network spectra. 8 network snapshots captured from 4 Aug 2002 to 30 Aug 2002, data from [16]. Only 600 largest eigenvalues computed and plotted for the last snapshot in part (h), showing principal eigenvalue and perimeter of bulk distribution.

To explore the modular organization of these networks, we explore the eigenvalue spectra of temporal snapshots of the peer-to-peer Gnutella file sharing network (data from [16]). The data represents a sequence of 8 snapshots of the P2P Gnutella network, collected in August 2002 (from 4 Aug 2002 to 30 Aug 2002, smallest network size [6301 nodes, 20777 edges] to largest network size [36682 nodes, 88328 edges]), with the nodes representing hosts and edges representing connections between hosts. Figure 6 shows the spectra for 8 temporal snapshots of this evolving network.

The results show a striking absence of large modules in all the 8 networks. The eigenvalue spectra show only one large eigenvalue well separated from the eigenvalue cloud, distinctly showing that there is no significant modularity present in the network. If there is any modularity present, we surmise that it is local; i.e., the size of the module is insignificant as compared to the size of the system, and that the modularity is very weak so as to be rendered undetectable by the spectral approach. As opposed to this signature, if there was any significant modularity present, the spectrum would have shown more than one eigenvalue well separated from the eigenvalue cloud. This was a very surprising result. One principle driving modularity in P2P systems could be that users on the P2P network are likely to have specific file sharing or information needs and exercise freedom in connecting to other users. Thus, an expected trend could be that modularity emerges in the network, even with the possibility that it is transient. Thus, it is remarkable that the evolving Gnutella P2P network (at least over a month of observations) shows a distinct absence of modularity. The random nature of user connections that is used as a model for the Gnutella network may explain the result. This finding has implications for P2P system design and performance. We note that the non-scalability of existing P2P Gnutella architecture, its reported mismatch with the underlying Internet topology, and new strategies for designing scalable and robust P2P systems has been the topic of much research [15]. Our analysis shows that these can be related to the finding that existing self-organized P2P systems appear to be non-modular, and that modularity and hierarchical organization are considered essential organizing principles in self-organized systems that maintain scalability of the system.

#### Social networks

**Figure 7:**
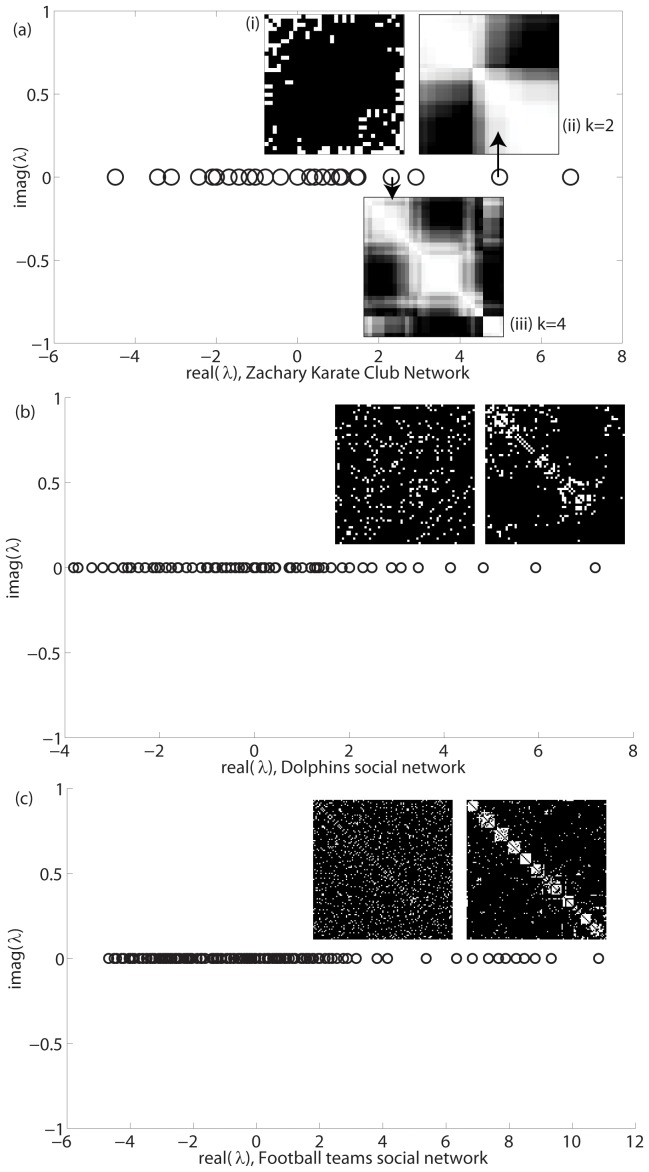
Social network spectra for benchmark test cases. Eigenvalues are all real because the networks are undirected. (a) Spectrum of Zachary Karate Club network data of 34 nodes from [20]. Inset (i) shows original adjacency matrix. Insets (ii) and (iii) show 2 dimensional and 4 dimensional approximations of original matrix. (b) Spectrum of Dolphins social network of 62 dolphins from [17]. (c) Spectrum of American Football Teams social network from [19]. Left inset shows unordered original network data. Right inset shows the same network, where rows and columns have been reordered using algorithm in [14] to show modules along the main diagonal.

As opposed to many technological and biological systems, social network data is usually available on smaller scales; i.e., the sizes of social networks are smaller in nature. Due to their small size, detailed study of community structure is possible, and some classic social networks with known modularity structure are often used as benchmark cases for modularity detection approaches and algorithms. Here, we explore the eigenvalue spectra of several social networks that are used as benchmark test cases. In all cases, the number of largest eigenvalues separated from the bulk distribution correctly predicts the known number of modules in the networks. Figure 7 shows the results for the well known Zachary Karate Club network, Dolphins social network [17], [18] and the American College Football data set [19].

The Zachary Karate Club network is one of the most studied social network data sets in the literature [1], [20]. Its small size and known partitions render it usable as “real world” test data for community detection techniques and algorithms. Members of a Karate club, 34 in number, split into 2 known communities follwing a disagreement between 2 leaders in the group. The split into 2 groups is known and well-documented and other authors have also studied the network for its hierarchical structure, and have shown that the two sub-groups split into smaller communities, showing 4 communities at a second hierarchical level [1]. The eigenvalue spectrum clearly reveals this analysis. Figure 7(a) shows the eigenvalue spectrum with two largest eigenvalues (signifying the major split of the network into two main parts), and two more clustered eigenvalues separated from the bulk distribution (signifying the second hierarchical level with 4 communities). The inset (i) shows the original adjacency matrix. In insets (ii) and (iii), following the algorithm presented in [14], we have produced 2 dimensional and 4 dimensional approximations of the original adjacency matrix by preserving, respectively, 2 and 4 largest eigenvalues and associated eigenvectors. It can be clearly seen in the lower dimensional approximations that at 2 eigenvalues the network shows 2 communities, and at 4 eigenvalues, these larger communities have split into two each, showing 4 communities. In both approximations, some nodes fall in overlaps between communities. These results correctly reveal the *exact* known partitions in the network [1].

The undirected Dolphin social network [17], [18] is a widely-cited example in the community detection literature. A group of dolphins were observed over a period of time, after which the groups split into two following the disappearence of a few members that were on the boundary of the group. Nodes in the social network represent these dolphins, and edges represent regular social contact. The group has a known community structure, with two well separated groups into which the bigger group split, and the larger of the groups showing further submodules. The spectra shows a clear indication of this hierarchical structure [Fig. 7(b)], with two largest eigenvalues being followed by a cluster of 3–4 eigenvalues that are well separated from the bulk distribution. We have previously studied the hierarchical structure of this network using an alternate spectral approach in detail and the results are in [14]. The insets show the unordered network data, and reordered organization to show the modules. The hierarchical structure is clearly visible.

The undirected American College Football dataset [19] is another well known dataset with a known community structure. There are 115 college teams that all play against each other. However, they are organized in “conferences” such that more frequent games occur between teams in a conference than between teams belonging to separate conferences. The known community structure corresponds to 12 conferences into which the teams are divided. The spectrum distinctly shows 12 large eigenvalues well separated from the bulk distribution [Fig. 7(c)], and the insets show the original unordered data, and the reordered matrix to show the 12 teams. We have performed the reordering using the algorithm described in [14].

#### Structural brain networks

Brains have fine-scale regular structure, to a first approximation, with high connectivity between nearby neurons [21]. Connectivity decreases as distance between neurons increases. Paradoxically, the brain also shows large scale specialization, with specific regions devoted to specific sets of functions [2]–[5]. The assumption is that these areas, often termed *modules*, are tightly connected together to perform certain functions and are only sparsely connected to other specialized areas. This assumption and experimental evidence supporting modularity is in apparent contradiction with the observed fine scale (nonmodular) homogeneity. Thus, reliably characterizing the structure of the brain is an unsolved problem. In a future paper, we use the methods reported here to resolve this contradiction between and simultaneous presence of fine scale regularity and large scale modularity in brain networks [22].

**Figure 8:**
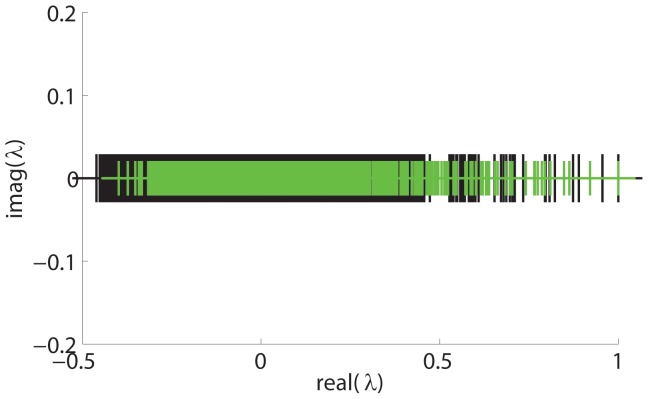
Human brain structural connection network spectrum. 998 node structural connection network spectrum (black pluses) compared with a 6 level 1024 node hierarchical network spectrum (green pluses). Both spectra have been divided by the corresponding largest eigenvalue to allow for superimposition to show relative scaling and relationship to each other.

Here, we examine the eigenvalue spectrum of a human brain structural connectivity network. The human brain structural network was obtained from [23]. They performed high resolution diffusion spectrum MRI (DSI) of the human cortex. They then defined 66 cortical regions with anatomical landmarks. Each of these 66 cortical regions was then individually subdivided into 998 regions of interest (ROIs). Weighted undirected networks were produced at two resolutions, a fine resolution network of 998 nodes and a coarse resolution network of 66 nodes. We study the spectrum of the fine scale 998 node network here. Figure 8 shows the results. The spectrum shows clusters of leading eigenvalues and a bulk distribution. However, the distinction between these clusters is highly smoothed, meaning that there are no clear gaps visible between hierarchical levels, with the leading eigenvalues gradually merging into the bulk distribution.

In the previous sections of *Results* on hierarchical networks, we showed that the above signature is typical of a network in which if it has many levels of hierarchy with the finer level hierarchical levels having smaller modules, then some of the leading eigenvalues get subsumed into the bulk distribution and hence cannot be detected. Thus, we generate a typical 6-level hierarchical modular network with 64 modules of size 16, followed by 32 modules of size 32, 16 modules of size 64, 8 modules of size 128, 4 modules of size 256, and 2 modules of size 512, following the stochastic block model network generation model described in the section *Spectrum of hierarchically modular networks* [12]. We set a low 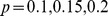 and high 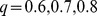. However, generating these hierarchical networks using the simple stochastic block model form does not produce a spectrum that matches the brain connectivity spectrum, although it successfully explains why the finer hierarchical levels with smallest module sizes cannot be detected.

Since the stochastic block model form is too simple to capture the properties of real world networks, we used a more sophisticated modified version of the stochastic block model, discussed in [12], [22], [24] to generate hierarchical networks with the above stated parameters. In this modified network generation model, instead of placing random blocks in successive hierarchical levels, we generate a hierarchical network by starting with a perfect modular network (fully connected modules) and successively rewiring it with decreasing probabilities at subsequent hierarchical levels. Figure 8 we superimpose (green pluses) the spectrum of this hierarchical network, 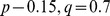, over the human brain network spectrum. The close match between the two spectra is clearly visible. This finding explains the observation that in human brain networks, 5 to 6 large modules are frequently detected [4], [5], [22], [23]. As shown by the spectrum, this large scale modularity is visible via the 5–7 leading eigenvalues in clusters separated from the bulk distribution. However, on finer scales the network appears nonmodular because the finest level modules are impossible to detect – the corresponding eigenvalues are subsumed in the bulk distribution. We note here that this undetectability, in an algorithm-independent manner, and notwithstanding the weakness inherent in any detection algorithm, may be a characteristic property of a natural system in which there are multiple hierarchical levels present. In such a system, the smallest sized modules may be so small as compared to the system size so that intermodular connection probabilities approaching close to the intramodular connection probabilities at that level cannot be avoided, thus making the modular structure extremely weak and rendering it undetectable.

## Discussion

In this paper, we address the problem of characterizing the hierarchical modularity of a network. The main results are a set of methods in which we develop a spectral approach to characterize the hierarchical modularity of networks in an algorithm independent manner, establish conditions for the detectability or undetectability of modularity in networks, and illustrate these results with synthetic and real world test cases. Our main results are:

- We derive the spectrum of hierarchically modular graphs generated using a stochastic block model form. Specifically, using theorems from random matrix theory, we derive the mean expected values for the set of largest eigenvalues of the adjacency matrix of a hierarchically modular graph. We show that hierarchical modularity of this model can be fingerprinted using the spectrum of its largest eigenvalues and gaps between clusters of closely spaced eigenvalues that are well separated from the bulk distribution of eigenvalues around the origin.
- We establish the limits of “how” modular a real world system is through a study of the properties of the spectrum and its distribution. The spectrum of modular networks with no hierarchy is shown to be a special case of our more general results, and some known results on the spectrum of modular networks are thus reproduced within our common framework for characterizing network modularity in general.
- We establish the limits of detection of hierarchical modularity and modularity as permitted by the spectral approach; i.e., given the amount or degree of modularity, we determine how much of this modularity can be (or cannot be) detected using the spectral approach. We empirically show that when probability parameters for instantiating edges in networks are varied, there is a threshold set by the probabilities and the limits of the bulk distribution of eigenvalues around the origin beyond which hierarchy and modularity cannot be detected even if weakly present.

As noted in the recent work of [11], spectral signatures of modularity detection are optimal in the sense that no other method can detect modularity in a regime where the spectral methods fail. This establishes that the results we present in this paper on the limits of modularity detection are general in the sense that if the spectral fingerprint fails to detect weak forms of modularity in a network, then any of the current spectral based methods and algorithms used for modularity detection are likely to be unable to detect it.

We studied the eigenvalue distributions of some technological, social, and biological networks, and showed that the spectrum can successfully capture information about the modular, hierarchically modular, or even non modular structure of real world networks (as for the case of P2P networks). This detection does not rest on the specific assumptions of any modularity detection algorithm. A study of the real world networks also revealed that the simple stochastic block model is insufficient to capture properties of real world networks (such as brain networks), and more sophisticated models are needed to capture these properties. In future work, analytical examination of the spectral properties of more sophisticated network models will allow us to address the problem of modularity and hierarchy detection more robustly.

Very importantly, a detailed study of structural brain network spectra revealed that, notwithstanding the weakness inherent in any detection algorithm or approach, the undetectability of modules can be a characteristic property of a natural system in which there are multiple hierarchical levels present. In any such natural system, the smallest sized modules at the finest hierarchical levels may be very small as compared to the system size. Thus, an unavoidable condition in such a situation is that the intermodular connection probabilities approach very close to the intramodular connection probabilities (since the nodes within the smallest module *have to* connect to nodes from other such small modules, thus making the modular structure extremely weak and rendering it undetectable.

## Methods

Here we present some old, classically known results that we use to derive our new results in this paper.

### Network Representation and Adjacency Matrix

Throughout the paper, in a network (or graph) 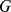, a node represents a component of the system, and edges represent structural or functional relationships between the nodes. In an adjacency or connection matrix representation of 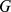, denoted by 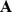, the rows/columns represent the nodes and entries 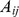 represent the weights of the edges. If the graph is undirected, 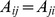, leading to a symmetric adjacency matrix. A symmetric matrix always has real eigenvalues. If the graph is directed, then 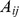 signifies an edge going from node 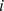 to node 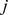, and in general, 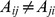. A directed graph produces an asymmetric adjacency matrix with complex eigenvalues. We consider both asymmetric and symmetric matrices in deriving the approximate spectra of networks in this work, that represent directed and undirected graphs, with 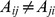, and 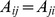, respectively.

We establish the main results in this paper using the following theorems from random graph theory and random matrix theory.

### Spectrum of an Uncorrelated Random Graph

An Erdös-Renyi uncorrelated random graph is a graph of 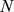 nodes where the probability for any two pairs of vertices in the graph being connected is the same, 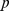, and these probabilities are independent variables [8]. Thus, the entries have a common expectation (mean) value of 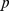 with a variance of 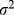. The main classically known results about the spectrum of uncorrelated random graphs that are of relevance in the present work relate to the distribution of its eigenvalues (see Ref. [25]). First, as the number of nodes 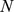 grows, the principal eigenvalue (the largest eigenvalue 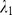) grows much faster than the second eigenvalue with 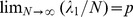 with probability 1, whereas for every 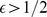, 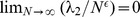. The same relationship holds for the smallest eigenvalue 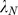. For every 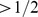, 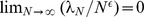. Thus, if 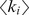 is the average degree of a vertex, then the largest eigenvalue 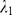 scales as 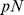 and the other eigenvalues 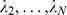 scale as 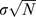.

These results were presented in a more detailed form in [26] for undirected graphs or symmetric random matrices. In [26], a matrix 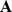 was considered with independent random variables 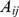, 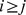, bounded with a common bound 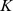. The common bound implies that all 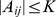 for all 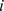 and 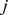. For 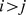, the 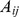 were considered to have a common expectation value 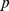 and variance 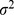, and the expectation value of 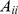 was considered to be 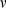. Then, 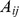 for 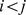 was defined by 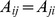. The numbers 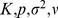 are held fixed as 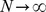, and the mean expected values of the largest eigenvalue and the limits of the bulk distribution of the other eigenvalues were studied.

**Figure 9:**
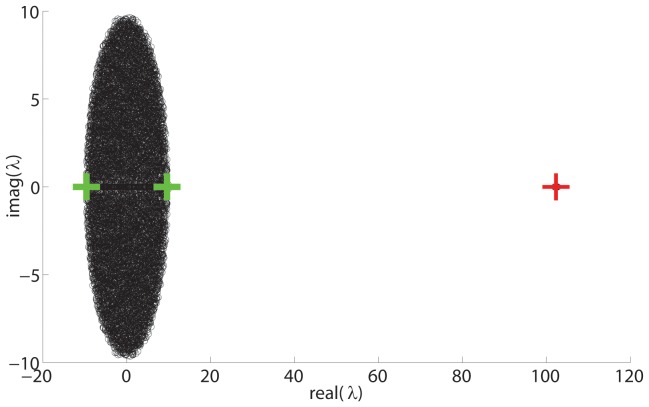
Uncorrelated random graph spectrum and analytic prediction of eigenvalue distribution. Red plus shows analytic prediction of largest eigenvalue; green plusses show the limits 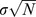 of the rest of the distribution. Spectra of 100 graphs are plotted, 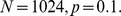

From the results of [26], if 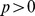 then the distribution of the largest eigenvalue of the random symmetric matrix 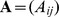 can be approximated in order 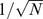 by a normal distribution of expectation
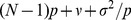
(18)and variance 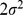. Further, with probability tending to 1, as 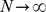,



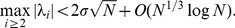
(19)If the expectation value of the diagonal elements 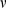 is 0, as is the case with adjacency matrices of graphs with no self-connections, then the second term in Eq. (18) vanishes. If 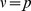, as is the case with adjacency matrices of graphs with self connections allowed, then the first two terms become 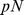. If the variance is restricted to be small, then the contribution of the third term in Eq. (18) is small. Then, in general, the leading term 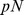 (for networks with self-connections allowed) or 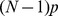 (for networks with no self-connections) makes the biggest contribution to the largest eigenvalue. Figure 9 shows the eigenvalue distribution of 100 random graphs with 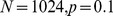, and analytical predictions from Eqs (18) and (19).

If the common expectation value 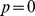, then
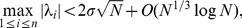
(20)implying that all the eigenvalues will be contained by the limits specified in Eq. (20). We note here that the for directed graphs with asymmetric matrices, this bound is known to be 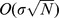, because in an undirected graph each value appears twice due to the condition 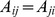.
